# Fc receptor-like 3 (*−169T>C*) polymorphism increases the risk of tendinopathy in volleyball athletes: a case control study

**DOI:** 10.1186/s12881-018-0633-6

**Published:** 2018-07-18

**Authors:** José Inácio Salles, Lucas Rafael Lopes, Maria Eugenia Leite Duarte, Dylan Morrissey, Marilena Bezerra Martins, Daniel Escorsim Machado, João Antonio Matheus Guimarães, Jamila Alessandra Perini

**Affiliations:** 1 Research Division, National Institute of Traumatology and Orthopaedics, Avenida Brasil, 500, Rio de Janeiro, RJ 20940-070 Brazil; 2 Federation International de Volleyball (FIVB) - Coach Commission, Rio de Janeiro, Brazil; 3 Research Laboratory of Pharmaceutical Sciences, West Zone State University, Rio de Janeiro, Brazil; 4 Centre for Sports Exercise Medicine, Queen Mary University of London, London, UK; 5 Program of Post-graduation in Public Health and Environment, National School of Public Health, Oswald Cruz Foundation, Rio de Janeiro, Brazil

**Keywords:** Fc receptor-like 3, Forkhead box P3 gene, Single nucleotide polymorphism, Tendinopathy, Volleyball athletes

## Abstract

**Background:**

Tendinopathy pathogenesis is associated with inflammation. Regulatory T (Treg) cells contribute to early tissue repair through an anti-inflammatory action, with the forkhead box P3 (FOXP3) transcription factor being essential for Treg function, and the FC-receptor-like 3 (FCRL3) possibly negatively regulating Treg function. *FCRL3 –169T>C* and *FOXP3 –2383C>T* polymorphisms are located near elements that regulate respective genes expression, thus it was deemed relevant to evaluate these polymorphisms as risk factors for tendinopathy development in athletes.

**Methods:**

This case-control study included 271 volleyball athletes (146 tendinopathy cases and 125 controls) recruited from the Brazilian Volleyball Federation. Genotyping analyses were performed using TaqMan assays, and the association of the polymorphisms with tendinopathy evaluated by multivariate logistic regression.

**Results:**

Tendinopathy frequency was 63% patellar, 22% rotator cuff and 15% Achilles tendons respectively. Tendinopathy was more common in men (OR = 2.87; 95% CI = 1.67–4.93). Higher age (OR = 8.75; 95% CI = 4.33–17.69) and more years of volleyball practice (OR = 8.38; 95% CI = 3.56–19.73) were risk factors for tendinopathy. The *FCRL3 –169T>C* frequency was significantly different between cases and controls. After adjustment for potential confounding factors, the *FCRL3 –169C* polymorphism was associated with increased tendinopathy risk (OR = 1.44; 95% CI = 1.02–2.04), either considering athletes playing with tendon pain (OR = 1.98; 95% CI = 1.30–3.01) or unable to train due to pain (OR = 1.89; 95% CI = 1.01–3.53). The combined variant genotypes, *FCRL3 –169TC* or *–169CC* and *FOXP3 –2383CT* or *–2383TT*, were associated with an increased risk of tendinopathy among athletes with tendon pain (OR = 2.24; 95% CI: 1.14–4.40 and OR = 2.60; 95% CI: 1.11–6.10). The combined analysis of *FCRL3 –169T>C* and *FOXP3 –2383C>T* suggests a gene-gene interaction in the susceptibility to tendinopathy.

**Conclusions:**

*FCRL3 –169C* allele may increase the risk of developing tendinopathy, and together with knowledge of potential risk factors (age, gender and years playing) could be used to personalize elite athletes’ training or treatment in combination with other approaches, with the aim of minimizing pathology development risk.

## Background

The viscoelastic properties of tendons are fundamental for transmitting the force generated by muscle for sport performance. However, the tissue deformation during repetitive and continuous stress makes the tendons susceptible to injury [1]. High-level volleyball players have an inherently high training load and the constant repetition of technical movements may increases the risk for tendinopathy [2].

Historically, tendinopathy has been considered an inflammatory disease, nevertheless a failed healing response of the tendon is regarded as being the main disruption [3, 4]. Previous analysis of tissue samples with tendon pathologies has shown collagen fiber degeneration and disorientation, hypercellularity, angiogenesis and decrease in inflammatory cells [5–7]. In addition, further evidence has suggested that tendons submitted to repetitive mechanical stress and its damage to stromal tissues plays a critical role in the immune system response to regeneration [8]. Thus, the influx of immune cells and their subsequent cytokine production and the critical interactions with resident tenocytes were determinants of the inflammatory effect on the tendon repair or degeneration [9]. Recently, several studies with animal models and tissue samples of tendinopathy patients have reinforce that the immune cells play a key role in the pathophysiology of this disease. [4, 10, 11]. Moreover, other studies have exposed the presence of T lymphocytes in tendinopathic tissue samples and indicated that this cell population may be more immunologically active than was previously thought [11–13].

CD4 + Foxp3+ regulatory T cells (Treg) are a subset of T lymphocytes that mediate an inhibitory effect on immune activity by suppressing the proliferation and function of effector T cells [14, 15]. The forkhead box P3 (FOXP3) is a transcription factor that plays an essential role in the function of Treg cells, in regulating the immune response and maintaining immune tolerance [16]. The FOXP3, encoded by the gene with the same name, is located in chromosome Xp11.23, and consists of 11 exons and encode a 431 amino-acid protein [17]. Polymorphisms in *FOXP3* gene may interfere in the suppressive function of Treg cells, lead to immune system instability, and hence, to the development of disease [18, 19]. The *FOXP3 –2383C>T* (rs3761549) polymorphism is located in the first intron, close to the *FOXP3* promoter region, and has been associated with susceptibility to autoimmune diseases [20–22]. These results have suggest a discussion about the Treg cell functions in relation to the pathogenic mechanisms of tendinopathy.

Since the Treg cells maintain immunological tolerance and prevent autoimmune and inflammatory diseases [14, 23], understanding of the genes involved in these pathways is essential for a better understanding of the pathological mechanisms. In this context, Fc receptor-like 3 (FCRL3) is a glycoprotein of the immunoglobulin receptor superfamily, expressed in Treg cells that may play a role as a negative regulator of Treg function [24–26]. The FCRL3, encoded by the gene with the same name, is located in chromosome 1q21–23, and has a functional polymorphism in the promoter region (*FCRL3 -169T>C*, rs7528684) that changes promoter activity and consequently alters nuclear factor-κB (NFκB) binding [27]. Moreover, *FCRL3 –169C* polymorphism has been associated with higher expression of FCRL3 in Treg cells [24, 27]. Due to the importance of their signaling domains in various immune cell types, the *FCRL3* gene probably modulates immune cell functions, and affects signaling pathways.

We hypothesized that polymorphisms in *FCRL3* and *FOXP3* genes may influence the onset and/or the progression of tendinopathy. The main aim of this study was to investigate the contribution of *FCRL3 –169T>C* and *FOXP3 –2383C>T* polymorphisms as risk factors for tendinopathy development in volleyball athletes, as well as their association with tendinopathy symptoms and sports activities.

## Methods

### Study design

**Fig. 1:**
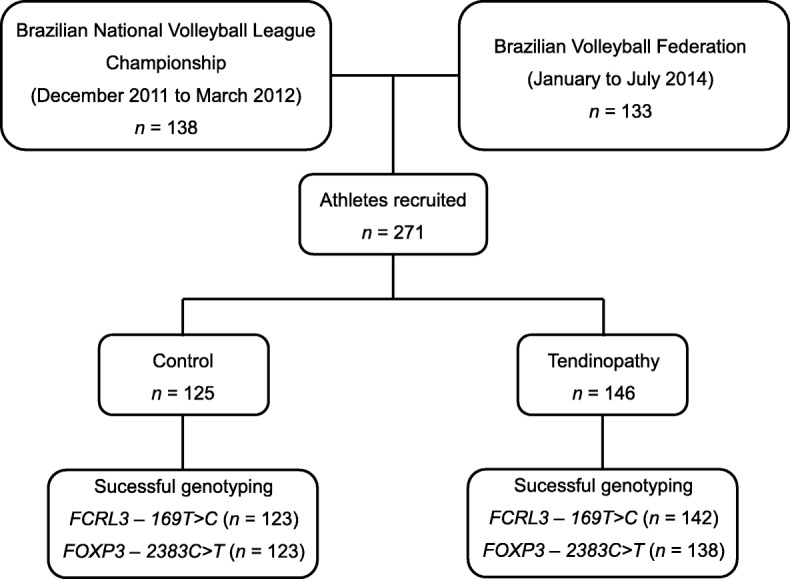
Flowchart of the study population

The study protocol was approved by the Human Ethics Committee of the Brazilian National Institute of Traumatology and Orthopedics (Protocol number 0037.0.305.000/2011 and 17373613.8.0000.5273/2013). Two hundred and seventy one athletes recruited via the Brazilian Volleyball Federation in Rio de Janeiro, Brazil. A study flowchart (Fig. 1) describes the athlete recruitment period (tendinopathy cases and controls), and the number of samples successfully genotyped for each polymorphism.

Inclusion criteria were volleyball players from the Brazilian Volleyball Federation. All participating athletes or their parents/legal guardians provided written informed consent and answered a questionnaire detailing demographics, sports activities, medical history, personal tendon injury and painful symptoms. The questionnaires were personally administered in two periods, December 2011–March 2012 and January 2014–July 2014, during training and the competition. The questionnaires included questions about ethnicity, self-identified according to the classification scheme adopted by the Brazilian Census (http://www.ibge.gov.br), which relies on self-perception of skin color. Accordingly, individuals were distributed in three “race/color” groups: *branco* (white, *n* = 103), *pardo* (meaning brown, here denoted as intermediate, *n* = 7), and *preto* (black, *n* = 22). One hundred thirty-nine athletes (51.3%) declined to give information about ethnicity.

The athletes were separated into cases (*n* = 146) and controls (*n* = 125) group (Fig. 1), according the presence or absence of tendinopathy clinically diagnosed by medical practitioners and confirmed with magnetic resonance image examination (MRI). The confirmatory MRI was performed during the Volleyball National Championship (December 2011 to March 2012) and the training of Brazilian Volleyball Federation carried out from January through July 2014. All diagnoses were confirmed by two blinded radiologists. As described in our previous studies [28, 29], the chronic tendinopathy diagnostic were (i) progressive pain related to training in the last 6 months and during clinical examination; and at least one of the following criteria: (ii) palpable nodular thickening over the tendon; (iii) tenderness on tendon palpation; (iv) history of swelling over the tendon area. The control group consisted of athletes with absence of tendinopathy history in any joint and who present no previous diagnosis of tendinopathy.

### Genotyping of polymorphisms

Genomic DNA was obtained from saliva samples as previously described [28]. The genotyping analyses of *FCRL3 –169T>C* (rs7528684) and *FOXP3 –2383C>T* (rs3761549) polymorphisms were performed using a TaqMan allelic discrimination assay obtained from Applied Biosystems (C_1741825_10 and C_27058744_10, respectively). For all polymorphisms real-time polymerase chain reaction (PCR) reactions were performed on a 7500 Real-Time System (Applied Biosystems, Foster City, CA, USA), and the genotypes were then determined directly.

### Statistical analysis

The sample size was calculated using Epi Info 7, version 7.1.3. (http://wwwn.cdc.gov/epiinfo/ html/downloads.htm) to detect a difference between case and control groups, assuming an odds ratio of 2.0 with a power of 0.8 and 5% type I error.

The Student’s *t*-test was conducted to compare continuous variables between tendinopathy cases and controls and were expressed as the mean ± standard deviation (SD). Chi-square (χ2) test or Fisher’s exact test, when applicable, was applied to compare differences in nominal data, as well as for the statistical analysis of the distribution frequencies of genotypes and alleles between the two groups. Additionally, Hardy-Weinberg equilibrium (HWE) was calculated by the χ2 test for goodness-of-fit. *FCRL3 –169T>C* and *FOXP3 –2383C>T* allele frequency and genotype distribution were derived by gene counting.

Multivariate logistic regression analyses were performed to identify possible confounding factors in the associations between polymorphisms and tendinopathy or between polymorphisms and tendinopathy features, which was estimated by the odds ratio (OR) with a 95% confidence interval (95% CI). The difference was statistically significant when *p* < 0.05. All analyses were performed using the Statistical Package for Social Sciences (SPSS Inc., Chicago, IL, USA), version 20.0.

## Results

**Fig. 2:**
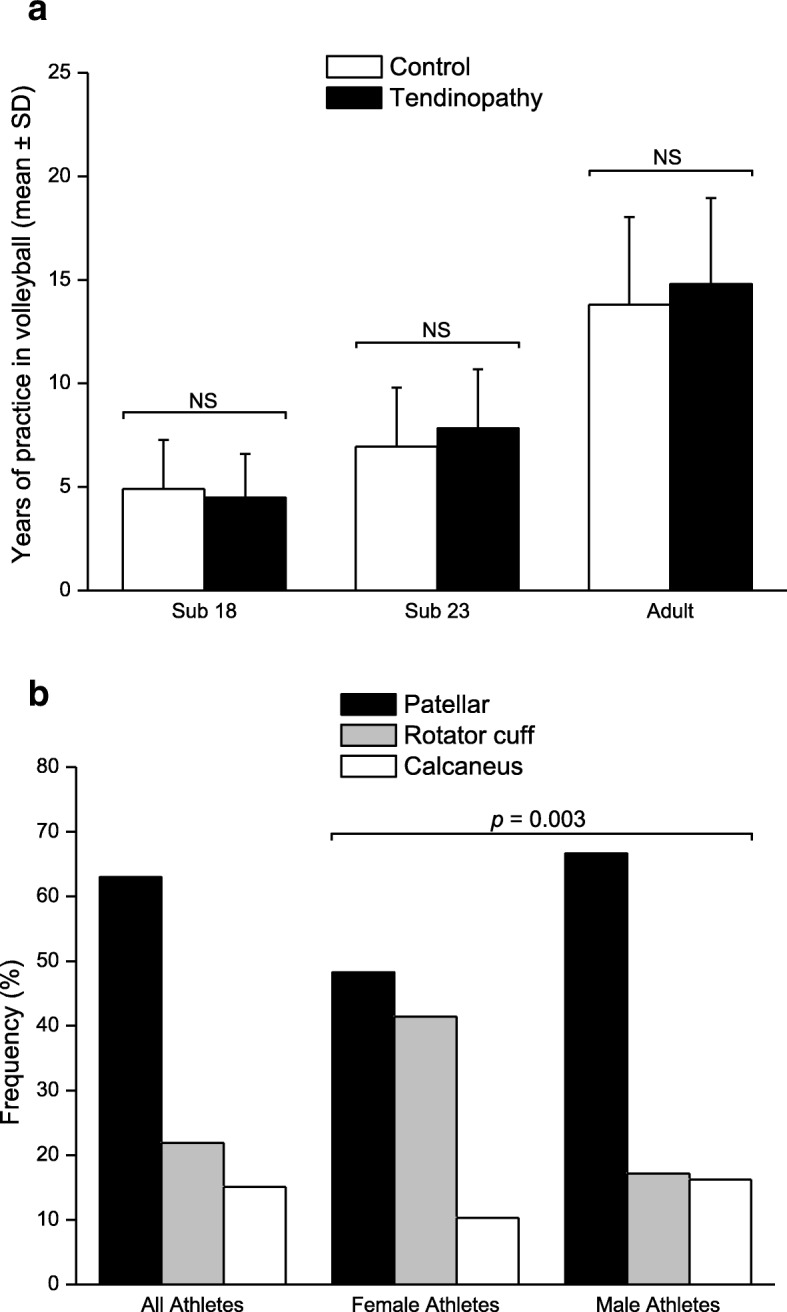
**a** Average time of practice in volleyball by age group in tendinopathy cases (*n* = 146) and controls (*n* = 125). Note: NS is not significant in Student T test (*p* > 0.05). **b** Distribution of the sites most affected by tendinopathy in Brazilian volleyball athletes (*n* = 146). *p* < 0.05 was obtained through the Chi-squared Test (Pearson *p*-value)

Table 1 presents the demographic, clinical and sport characteristics of the volleyball athletes. There were significant differences between the tendinopathy cases and controls with regard to mean age (26.86 ± 6.03 and 21.62 ± 5.39, respectively, *p* = 0.0001), average time of years of practice in volleyball (12.27 ± 5.35 and 8.27 ± 4.92, respectively, *p* = 0.0001), gender and tendinopathy clinical symptoms (tendon pain and away from training due pain). The evaluation of demographic and clinical characteristics revealed the athlete male gender (moderate risk), older age and higher years of practice in volleyball (very large risk) were risk factors for tendinopathy (Table 1). However, there were no significant differences between the two groups concerning the average time of practice in volleyball by age group (Student T test, Fig. 2a). The frequency of tendinopathy by tendon among elite volleyball athletes is shown in Fig. 2b. There was a significant gender difference among to the affected tendon type (*p* = 0.003, χ2 test).

**Table 1:** Characteristics of the volleyball athletes (*n* = 271)

Variables	Controls (*n* = 125)	Tendinopathy (*n* = 146)	*p*-value^a^	OR (95% CI)
Age group	*n* (%)			
Sub - 18	47 (37.6)	14 (9.6)	< 0.001	1^b^
Sub - 23	40 (32.0)	33 (22.6)		2.77 (1.30–5.89)
Adult	38 (30.4)	99 (67.8)		8.75 (4.33–17.69)
Gender				
Female	52 (41.6)	29 (19.9)	< 0.001	1^b^
Male	73 (58.4)	117 (80.1)		2.87 (1.67–4.93)
Ethnicity^c^				
White	67 (74.4)	36 (85.7)	0.41	1^b^
Intermediate	5 (5.6)	2 (4.8)		0.74 (0.14–4.03)
Black	18 (20.0)	4 (9.5)		0.41 (0.13–1.31)
Years of practice in volleyball				
0–5	45 (36.0)	17 (11.6)	< 0.001	1^b^
6–10	46 (36.8)	40 (27.4)		2.30 (1.14–4.64)
11–15	22 (17.6)	51 (34.9)		6.14 (2.90–12.98)
> 15	12 (9.6)	38 (26.1)		8.38 (3.56–19.73)
Declared preference				
Right	120 (96.0)	141 (96.6)	0.53	1^b^
Left	5 (4.0)	5 (3.4)		0.85 (0.24–3.01)
Function				
Spiker	90 (72.0)	115 (78.8)	0.34	1^b^
Setter	22 (17.6)	22 (15.1)		0.78 (0.41–1.50)
Libero	13 (10.4)	9 (6.1)		0.54 (0.22–1.32)
Traumatic lesion				
No	85 (68.0)	88 (60.3)	0.23	1^b^
Yes	40 (32.0)	58 (39.7)		1.40 (0.85–2.31)
Tendon pain				
No	47 (37.6)	14 (9.6)	< 0.001	1^b^
Yes	78 (62.4)	132 (90.4)		5.68 (2.94–10.98)
Away from training due pain				
No	91 (72.8)	80 (54.8)	0.003	1^b^
Yes	34 (27.2)	66 (45.2)		2.21 (1.32–3.68)

Notes: *OR* odds ratio; *CI* confidence interval

^a^Chi-Square Test or Fisher’s exact test

^b^Reference group

^c^There are ethnicity information of 132 athletes

**Fig. 3:**
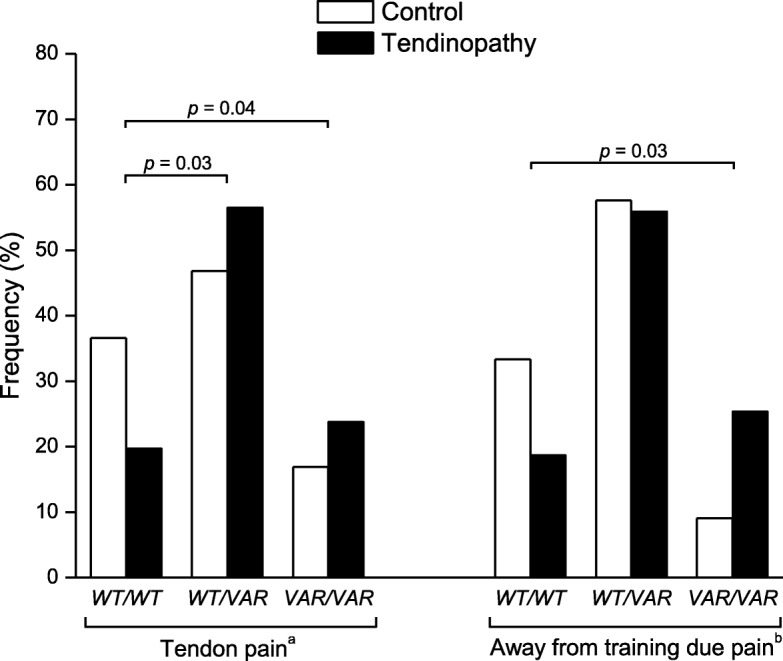
Combined analysis of the *FCRL3 –169T>C* and *FOXP3 –2383C>T* polymorphisms and the risk of developing of tendinopathy among athletes who present pain or who were away from training due pain. Notes: *WT/WT: FCRL3 -169TT and FOXP3 -2383CC. WT/VAR: FCRL3 -169TT* and *FOXP3 -2383CT; FCRL3 -169TT* and *FOXP3 -2383TT; FCRL3 -169TC* and *FOXP3 -2383CC* or *FCRL3 -169CC* and *FOXP3 -2383CC. VAR/VAR: FCRL3 -169TC* and *FOXP3 -2383CT; FCRL3 -169TC* and *FOXP3 -2383TT; FCRL3 -169CC* and *FOXP3 -2383CT* or *FCRL3 -169CC* and *FOXP3 -2383TT. p* < 0.05 was obtained through the Chi-squared Test (Pearson *p*-value) or Fisher’s exact test. ^a^The analysis for tendon pain was adjusted by age, years of practice in volleyball and gender. ^b^The analysis for away from training due pain was adjusted by age, years of practice in volleyball, gender and pain

The *FCRL3 –169T>C* and *FOXP3 –2383C>T* polymorphisms were in Hardy–Weinberg equilibrium. The allelic and genotypic frequencies and association’s analyses of both polymorphisms are summarized in Table 2. Athletes with tendinopathy showed a significant higher frequency of the variant allele *FCRL3 –169C* compared with the controls. After adjusting for confounding factors (age, years of practice in volleyball, gender and pain), evaluated in multivariate logistic regression models, *FCRL3 –169C* polymorphism was associated with a higher risk of tendinopathy. By contrast, no significant differences were detected in the *FOXP3 –2383C>T* polymorphism frequency between the two groups (Table 2). In addition, *FCRL3 –169T>C* polymorphism frequency was significantly different regards to tendon pain and were away from training due pain between cases and controls who presented these clinical symptoms complaints (Table 3). After adjusting for confounding factors, the *FCRL3 –169C* allele increases the risk (approximate 2-fold) of developing tendinopathy among athletes who present pain or were away from training due pain. Moreover, a combined analysis of the *FCRL3 –169T>C* and *FOXP3 –2383C>T* polymorphisms was performed to investigate if their interaction would increase the risk of developing of tendinopathy among athletes who present tendon pain or were away from training due pain (Fig. 3). It has been observed that relative to the combined wild-type genotype (*FCRL3 –169TT* and *FOXP3 –2383CC*), the combined variant genotype (*FCRL3 –169TC* or *–169CC* and *FOXP3 –2383CT* or *–2383TT*) were associated with an increased risk of developing tendinopathy among athletes who present tendon pain (WT/VAR: OR = 2.24; 95% CI: 1.14–4.40 and *VAR/VAR*: OR = 2.60; 95% CI: 1.11–6.10) or were away from training due pain (*VAR/VAR*: OR = 5.00; 95% CI: 1.12–22.30).

**Table 2:** Association analyses of the *FCRL3 –169T>C* and *FOXP3 –2383C>T* polymorphisms in tendinopathy cases compared with controls

*SNP*	Controls *n* (%)	Tendinopathy	*p*-value^a^	OR (95% CI)^b^
*FCRL3 -169T>C*	(*n* = 123)	(*n* = 142)		
*TT*	48 (39.0)	40 (28.2)		1^c^
*TC*	56 (45.6)	70 (49.3)	0.19	1.50 (0.86–2.59)
*CC*	19 (15.4)	32 (22.5)	0.04	2.02 (0.10–4.09)
*TC + CC*	75 (61.0)	102 (71.8)	0.08	1.63 (0.97–2.73)
*T*	152 (61.8)	150 (52.8)	0.04	1^c^
*C*	94 (38.2)	134 (47.2)		1.44 (1.02–2.04)
*FOXP3 -2383C>T*	(*n* = 123)	(*n* = 138)		
*CC*	86 (69.9)	97 (70.3)		1^c^
*CT*	30 (24.4)	37 (26.8)	0.86	1.09 (0.62–1.92)
*TT*	7 (5.7)	4 (2.9)	0.45	0.51 (0.14–1.79)
*CT + TT*	37 (30.1)	41 (29.7)	1.0	0.98 (0.58–1.67)
*C*	202 (82.1)	231 (83.7)	0.71	1^c^
*T*	44 (17.9)	45 (16.3)		0.89 (0.57–1.41)

Note: *SNP* single nucleotide polymorphism, *OR* odds ratio, *CI* confidence interval

^a^Chi-Square Test or Fisher’s exact test

^b^Adjusted by age, years of practice in volleyball, gender and pain

^c^Reference group

**Table 3:** Analysis of *FCRL3 –169 T > C* polymorphism frequency with regards to tendon pain and athletes who were away from training due pain in cases compared with controls

*FCRL3 -169T>C*	Controls *n* (%)	Tendinopathy *n* (%)	*p*-value^a^	OR (95% CI)
Tendon pain^b^				
	(*n* = 77)	(*n* = 128)		
*TT*	38 (49.3)	34 (26.6)		1^d^
*TC*	29 (37.7)	65 (50.8)	0.007	2.5 (1.32–4.74)
*CC*	10 (13.0)	29 (22.6)	0.010	3.24 (1.38–7.62)
*TC + CC*	39 (50.7)	94 (73.4)	0.002	2.69 (1.49–4.88)
*T*	105 (68.2)	133 (51.9)	0.002	1^d^
*C*	49 (31.8)	123 (48.1)		1.98 (1.30–3.01)
Away from training due pain^c^				
	(*n* = 33)	(*n* = 63)		
*TT*	15 (45.5)	15 (23.8)		1^d^
*TC*	15 (45.5)	37 (58.7)	0.09	2.47 (0.97–6.28)
*CC*	3 (9.0)	11 (17.5)	0.14	3.66 (0.85–15.90)
*TC + CC*	18 (54.5)	48 (76.2)	0.05	2.67 (1.09–6.54)
*T*	45 (68.2)	67 (53.2)	0.03	1^d^
*C*	21 (31.8)	59 (46.8)		1.89 (1.01–3.53)

Differences in sample sizes are due to available data from PCR amplification for each polymorphism

Note: *OR* odds ratio, *CI* confidence interval

^a^Chi-Squared Test or Fisher’s exact test

^b^The analysis for tendon pain was adjusted by age, years of practice in volleyball and gender

^c^The analysis for away from training due pain was adjusted by age, years of practice in volleyball, gender and pain

^d^Reference group

**Fig. 4:**
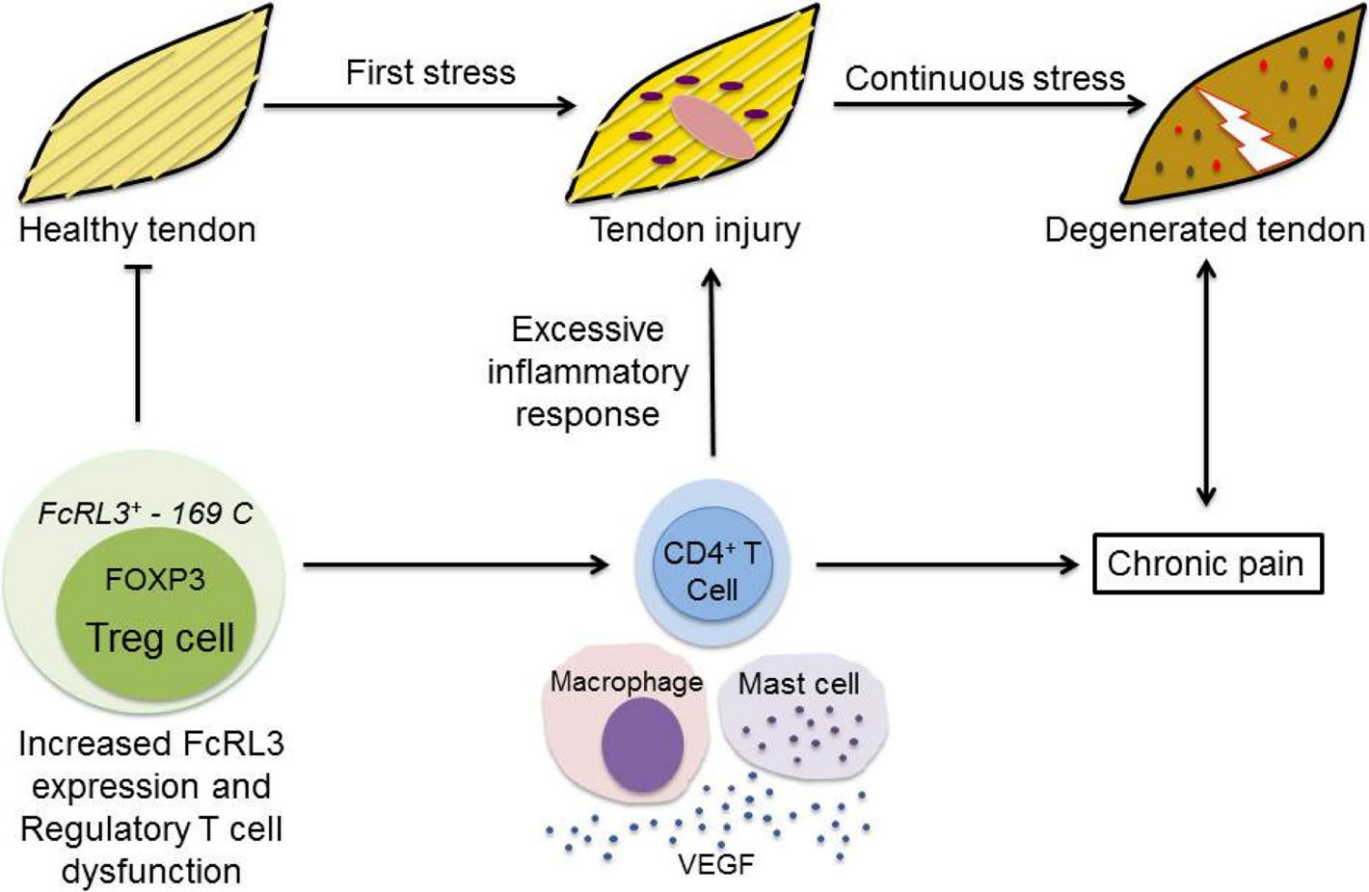
Hypothesis of the role of the *FCRL3-169C* allele and the inflammatory response of the injured tendon

Based on the results of this study and the previous ones, we propose a hypothesis for the role of *FCRL3 –169T>C* polymorphism in the tendinopathy development (Fig. 4).

## Discussion

High incidence of tendon overuse injuries prevails in elite volleyball athletes mainly because they have to go through many hours of practice [2]. Moreover, the biomechanical characteristics of the skills required in volleyball associated with the joint anatomy of the players are accepted as being the risk factors for overuse injuries [30]. Among the ball-related sports, volleyball is one of the types that cause a high rate of overuse injury by demanding repetition of similar movement patterns [31] with an professional volleyball attacker performing approximately 40,000 spikes a year [32]. This volume of spikes may be represented by the fact that from 8 to 20% of the injuries occurring in the shoulder [33] and nearly 45% of injuries in volleyball athletes are identified as patellar tendinopathy [34, 35]. In present study, tendinopathy in Brazilian volleyball athletes was more frequent in the knee (63% patellar) followed by the shoulder (22%). Moreover, female athletes presented a higher frequency of tendinopathy in the shoulder (41% in female versus 17% in male), corroborating the findings of a study unrelated to sport that the female gender was a risk factor for the development of rotator cuff disease [36]. Furthermore, Reeser and colleagues investigated risk factors for volleyball-related shoulder pain and dysfunction, and observed that female players showed lower simple shoulder test scores than male athletes [33].

Repetitive and strong physical activity, characteristic of elite athletes, contributes to excessive loading of tendons, promoting inflammation and pathological degeneration [4, 37]. Despite the development of physical qualities in high performance teams to reduce the tendinopathy risk, no standard that sufficiently compensates for the demands of the training has yet been established. Therefore, considering the high costs involved with overuse injuries in athletes, new strategies should be considered for the prevention of these injuries. Recently, studies based on identifying the DNA polymorphisms have been relevant in sports medicine investigation with the purpose of identifying the athletes most likely to develop lesions and thus suggesting individualized training to improve performance in sports [28, 29, 38–42].

As far as we know, the present study is the first study to focus on the possible contribution of the *FCRL3 –169T>C* and *FOXP3 –2383C>T* polymorphisms to the susceptibility to tendinopathy in elite athletes. The *FCRL3 –169T>C* polymorphism was associated with increased tendinopathy risk, either considering all cases, only athletes with tendon pain or those who were away from training due to pain. The *FCRL3 –169T>C* polymorphism changes promoter activity and consequently alters NFκB binding [27]. Therefore, the *FCRL3 –169T>C* polymorphism has previously been reported in association with rheumatoid arthritis [27, 43, 44], psoriasis vulgaris [22], neuromyelitis optica [45], multiple sclerosis [46] and endometriosis [21, 47]. Furthermore, in present study the combined variant genotypes of *FCRL3 –169T>C* and *FOXP3 –2383C>T* were also associated with an increased risk for developing tendinopathy among athletes who presented tendon pain or were away from training due to pain. The combined analysis of *FCRL3 –169T>C* and *FOXP3 –2383C>T* suggested a gene-gene interaction in the susceptibility to tendinopathy. The cumulative effect of interaction of *FCRL3 –169T>C* and *FOXP3 –2383C>T* polymorphisms has previously been demonstrated in the development of endometriosis [47]. The *FOXP3 –2383C>T* polymorphism may interfere in the FOXP3 factor, promoting Treg cell dysfunction and development of diseases [48].

In the early stages of tendinopathy, changes in tissue microenvironment and activation of the innate immune system contribute to inflammatory repair in tendons [4]. In an experimental animal model of chronic tendinopathy, mast cells and macrophages were recruited and released angiogenic growth factors, which stimulate the proliferation of new blood vessels [49]. Interestingly, the major angiogenic growth factor VEGF is not found in healthy tendons [50], but it is expressed in tendons in chronic degeneration [51]. Recently, our group investigated whether polymorphisms in *VEGF* and its receptor *KDR* genes could be correlated with susceptibility to tendinopathy. We described evidences that the polymorphisms in *KDR* might alter receptor activity, influence the angiogenic process and consequently contribute to inter-individual variation in the development of tendinopathy in volleyball athletes [29].

In addition to the recruitment of macrophages and mast cells, and increase in angiogenesis signals in the tendon injury microenvironment, the CD4+ T cell also migrated into tissue and released pro-inflammatory cytokines, including interleukin 2 (IL-2), enhancing the innate immune response for tendon repair [11]. However, the persistence of stimuli in the tendon over a long period may cause tissue degeneration by excessive inflammatory responses and lead to chronic pain [37]. In this context, we suggest that CD4 + Foxp3+ regulatory T cells (Treg) could modulate the function of effector T cells during tendon repair by regulating the expression of target genes in the inflammatory response. As already described in other pathological conditions, polymorphisms can modulate the function of Tregs, harming the immune response [24, 52]. In present study, we found that *FCRL3 -169C* allele was associated with increased tendinopathy risk. This allele increased *FCRL3* gene expression in Treg cells and promoted inflammatory response to a greater extent in by the CD4+ T cell generating higher levels of immune activation [24]. Thus, we proposed a hypothesis relative to the role of the *FCRL3 -169C* allele in Treg cell dysfunction preventing the tendon regeneration (Fig. 4).

The main limitation of this approach was that the present study did not collect information on ethnicity of all athletes. The high degree of admixture of different ethnic backgrounds (mostly Europeans, Africans and Amerindians) in the Brazilian population, poses special challenges to ethnic classification. The Instituto Brasileiro de Geografia e Estatística (IBGE) responsible for the official census of Brazil, has used only few pre-established color categories, which are based on self-classification (see Methods). However, there is poor correlation of self-reported race/color with genetic ancestry among Brazilians and this is based on a complex subjective phenotypic evaluation [53]. Therefore, it was not possible to apply adjustments for population stratification [54], in spite of no significant difference in ethnicity being observed between the tendinopathy athletes and the control group. The extrapolation of genetic data obtained from well-defined ethnic groups is not appropriate for application to Brazilians [53] and our data can be used in future studies to improve understanding of the risk factors involved in the development of tendinopathy in athletes.

Finally, our group has been developing studies with the purpose of identifying genetic characteristics that may clarify new therapeutic targets or personalized training programs to treat the disease or to avoid the development of tendinopathy in athletes. However, the information about magnitude of each variable is necessary to determine if the effect has an important role in practical and clinical decisions about applicability of the outcome [55]. In this study, *FCRL3* and *FOXP3* polymorphisms and male gender were associated with a moderate risk (approximate 2-fold), whereas athlete older age and higher years of practice in volleyball were associated with a higher risk (approximate 8-fold) of tendinopathy. The knowledge of the potential risk factors associated with tendinopathy may help to build models to use for diagnosing athletes susceptible to tendon injury and providing these athletes with additional personalized support.

## Conclusion

The cumulative effect of *FCRL3 –169T>C* and *FOXP3 –2383C>T* polymorphisms was associated with development of tendinopathy in Brazilian volleyball athletes, and this genetic knowledge together potential risk factors (age, gender and years of practice in volleyball) could improve the personalized training or treatment of athletes.

## Abbreviations

95% CI: 95% Confidence interval
FCRL3: FC-receptor-like 3
FOXP3: Forkhead box P3
HWE: Hardy-Weinberg equilibrium
IBGE: Instituto Brasileiro de Geografia e Estatística
IL2: Interleukin 2
KDR: Kinase insert domain receptor
MRI: Magnetic resonance image examination
NFκB: Nuclear factor-κB
OR: Odds ratios
PCR: Polymerase chain reaction
SD: Standard deviation
SNPs: Single nucleotide polymorphisms
Treg: Regulator T cells
VAR: Variant
VEGF: Vascular endothelial growth factor
WT: Wild-type
χ2: Chi-square
